# Chemotherapy-induced cognitive impairment is associated with decreases in cell proliferation and histone modifications

**DOI:** 10.1186/1471-2202-12-124

**Published:** 2011-12-09

**Authors:** Teresita L Briones, Julie Woods

**Affiliations:** 1 Department of Adult Health, Wayne State University, Detroit, MI 48202, USA; 2 Department of Biobehavioral Health Science, University of Illinois at Chicago, Chicago, IL 60612, USA

## Abstract

**Background:**

In this study, we examined the effects of cyclophosphamide, methothrexate, and 5-Fluorouracil (CMF) drug combination on various aspects of learning and memory. We also examined the effects of CMF on cell proliferation and chromatin remodeling as possible underlying mechanisms to explain chemotherapy-associated cognitive dysfunction. Twenty-four adult female Wistar rats were included in the study and had minimitter implantation for continuous activity monitoring two weeks before the chemotherapy regimen was started. Once baseline activity data were collected, rats were randomly assigned to receive either CMF or saline injections given intraperitoneally. Treatments were given once a week for a total of 4 weeks. Two weeks after the last injection, rats were tested in the water maze for spatial learning and memory ability as well as discrimination learning. Bromodeoxyuridine (BrdU) injection was given at 100 mg/Kg intraperitoneally 4 hours prior to euthanasia to determine hippocampal cell proliferation while histone acetylation and histone deacetylase activity was measured to determine CMF effects on chromatin remodeling.

**Results:**

Our data showed learning and memory impairment following CMF administration independent of the drug effects on physical activity. In addition, CMF-treated rats showed decreased hippocampal cell proliferation, associated with increased histone acetylation and decreased histone deacetylase activity.

**Conclusions:**

These results suggest the negative consequences of chemotherapy on brain function and that anti-cancer drugs can adversely affect the self-renewal potential of neural progenitor cells and also chromatin remodeling in the hippocampus. The significance of our findings lie on the possible usefulness of animal models in addressing the clinical phenomenon of 'chemobrain.'

## Background

The development of new chemotherapeutic agents and new regimens for breast cancer therapy has led to a reduced risk of recurrence and a higher rate of survival in this patient group. The majority of breast cancer survivors receive chemotherapy but unfortunately they also report chemotherapy-associated cognitive compromise. For example, in the first of a series of cross-sectional studies in women with early breast cancer, cognitive impairment was observed in 75% of patients after cytostatic treatment [1-3]. Although the results of subsequent cross-sectional trials assessing cognitive function during or after chemotherapy are less dramatic, all of them reported substantial cognitive impairment rates of 16% to 50%, suggesting detrimental cytostatic side effects on cognitive function [4-6]. The cognitive deficits reported in these studies range from very subtle to more severe and are observed in a wide range of brain functions, including memory, concentration, and speed of information processing, and can be noticed up to 10 years after completion of cytotoxic treatment.

Although the existence of chemotherapy-induced cognitive deficits has become almost universally recognized, other recently published studies raised some doubts on this phenomenon because they failed to confirm the adverse effects of chemotherapy on cognitive function [7-9]. The inconsistent findings reported to date make conclusions regarding a link between chemotherapy and cognitive impairment tenuous and underscore the need for further research in this area. The inconsistencies reported are most probably due to the inherent methodological limitations in studies involving human subjects, which include small samples, less than adequate controls, and most importantly failure to account for other factors (e.g., disease-related complications, stress, other co-morbidities) that could affect cognitive performance. The inherent methodological difficulties and ethical issues associated with conducting studies in clinical settings also lead to the inability to separately identify the effects of chemotherapy and malignancy itself on cognitive function. As well, the possible mechanisms of chemotherapy-related cognitive dysfunction remain poorly understood.

Currently, there are few animal studies that provide insight into the effects of chemotherapy on cognitive function. For instance, reports show that high-dose intravenously administered methotrexate reduce spontaneous activity and diminish startle response to loud noise or vibrissal stimulation [10,11] in male rats. Furthermore, high-dose intraperitoneal injections of methotrexate result in enhanced occurrence of seizures in mice and an impairment of long-term memory in a passive avoidance task [10]. Other studies that examined cyclophosphamide, doxorubicin, and 5-Fluorouracil given intraperitoneally also show that these drugs can cause a disruption of learning and memory across a variety of task such as water maze, avoidance conditioning, object recognition, as well as cue-specific and contextual fear conditioning tasks [12-16]. However, one study did not find any cognitive effects of treatment with 5-Fluorouracil on rat behavior [17] suggesting that even in animal models reported results on chemotherapy-induced cognitive impairment is far from being congruent, which may be attributed to the different drugs, dosing, and route of administration used.

**Figure 1:**
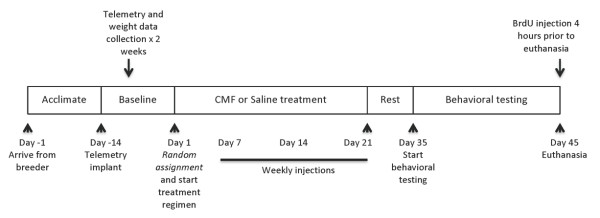
**Study Design**. Legends: CMF - cyclophosphamide, methothrexate, and 5-Fluorouracil.

To address the above issues we examined the effects of cyclophosphamide, methotrexate, and 5-fluorouracil (CMF) on a broad range of memory processes in female rats (Figure 1). CMF is an adjuvant chemotherapy widely used clinically [18] and together with the assessment of different memory processes, this study has the potential to serve as a model for examining whether the cognitive changes observed in breast cancer survivors are associated with chemotherapy rather than the malignancy itself. In addition, we measured activity level to determine the influence of fatigue, a symptom clinically associated with chemotherapy, on behavioral tasks performance.

Hippocampal cell proliferation has been implicated in learning and memory; and memory processing requires the coordinated effort of transcription factors and numerous enzymes and coregulators that modify and remodel chromatin structure, the covalent modification of histone tails (for review see [19]). Enzymes that regulate chromatin remodeling are known as histone acetyltransferases and histone deacetylases [20]. Thus, we also examined whether changes in hippocampal cell proliferation and histone modifications may be possible mechanisms involved in chemotherapy-related cognitive dysfunction.

## Results

### Chemotherapy Induces Fatigue and Weight Changes During Treatment

**Figure 2:**
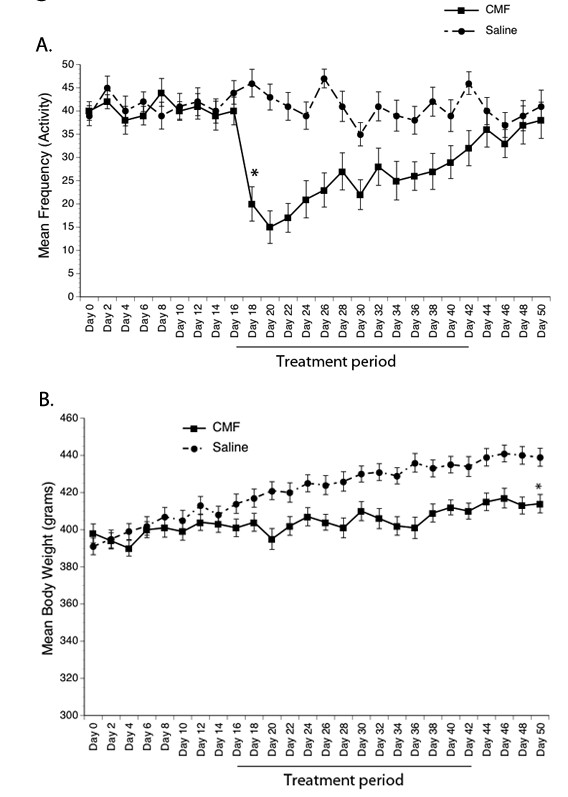
**Physical Activity and Body Weight**. General physical activity significantly decreased in the CMF group compared to the saline group during the treatment period but fatigue decreased once the drugs were discontinued (A). In contrast, no weight loss was seen in the CMF group during the treatment period (B). However, the CMF group failed to normally gain weight compared to the saline group although they remained healthy throughout the study and no overall reduction in food intake was observed. *p < 0.05.

Because of the possible confounding influence of other factors on cognitive performance, we examined whether chemotherapy can induce fatigue and weight loss. Our results showed a significant decrease in general activity during the treatment period but baseline levels were restored once the chemotherapy was discontinued (Figure 2A) suggesting that these cytotoxic drugs can cause fatigue symptoms. We also found a significant difference in weight between the chemotherapy- and saline-treated rats, in that those in the saline group continued to gain weight over the 4-week period compared to rats that received CMF (Figure 2B); however, the weight of rats in the chemotherapy group did not significantly differ from their baseline values. Although all rats in the study remained healthy throughout the experiment, repeated administration of CMF over the 4-week period resulted in the inability to gain weight even though no overall reduction in food intake was observed suggesting that the cytotoxic drugs might have an effect on gastrointestinal function.

### Chemotherapy Induces Memory Impairment

**Figure 3:**
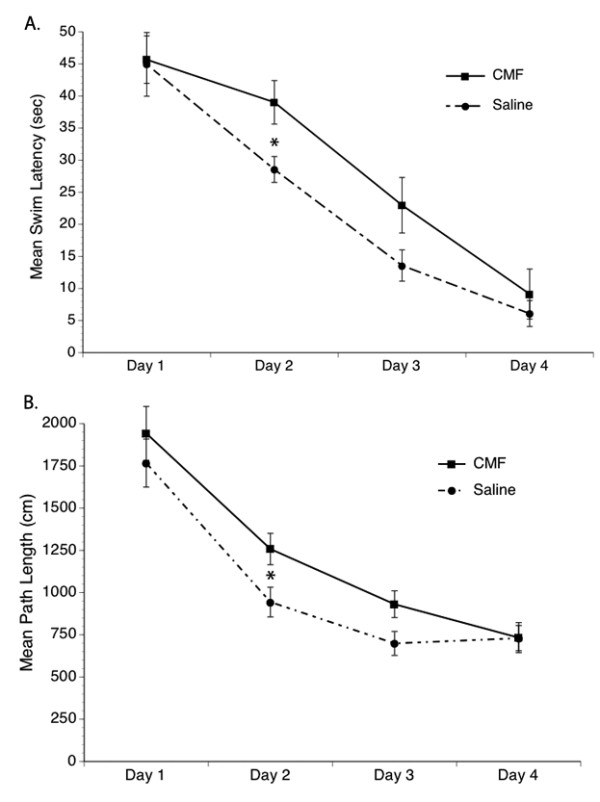
**Spatial Learning and Memory**. Significantly increased mean swim latency (A) and path taken to reach the goal (B) was seen in the CMF group. However, these rats eventually performed as well as the saline group in the spatial learning and memory tasks on the last day of testing. *p < 0.05.

Rats were tested in a series of water maze tasks designed to assess spatial learning and long-term memory of a fixed location. A significant within-subjects effect was seen for swim latency during the acquisition phase (first 4 days of testing) wherein all rats learned to perform the task efficiently over the four testing days (Figure 3). Significant group differences were seen in mean swim latency and path length in that the chemotherapy-treated rats did not perform the task as well as the saline-treated group until the third day of testing. But despite the longer mean swim latency and path length demonstrated by the chemotherapy-treated group in the beginning days of the trial, they eventually performed as well as the saline-treated groups. However, no significant treatment × time interaction was seen. No significant differences were seen in swimming speed in the chemotherapy- and saline-treated groups across the test days. These results suggest a chemotherapy-induced transient spatial learning impairment.

On the fifth day of behavioral testing, a probe trial was performed for 60 seconds wherein rats were placed in the water maze without the goal/platform. Time spent swimming in the quadrant of the pool where the goal/platform was located during the first 4 days of trial (correct quadrant) was divided by the time spent swimming in the other three quadrants of the pool (wrong quadrants). In the probe trial (recall phase) a significant difference was seen in that the chemotherapy-treated animals spent less time in the correct target quadrant area where the goal was previously located compared to the saline-treated group (Table 1). These results suggest that although the cytotoxic drugs have a transient effect on learning a simple task (acquisition), chemotherapy can induce some degree of persistent memory impairment as evidenced in the probe trial performance. However, no significant differences were seen in swimming speed suggesting the absence of motoric impairment. Furthermore, no significant differences were seen in the cued trial (Table 2) suggesting the absence of visual problems that can affect behavioral performance.

**Table 1:** Probe Trial.

	CMF	Saline
Time spent swimming in correct quadrant of the pool	32%*	66%

The CMF group spent significantly less time swimming in the correct quadrant of the pool during the probe trial compared to the saline group. Less amount of time spent swimming in the correct quadrant illustrates deficits in recalling learned information. * *p *< 0.05.

**Table 2:** Cue Learning Trial.

	CMF	Saline
Mean swim latency (sec)	16.05 ± 1.01	16.92 ± 0.92
Path Length (cm)	511 ± 29	488 ± 22

No significant differences were seen in the CMF-treated and saline groups when tested in the water maze with a visible pole suggesting the absence of visual impairments.

**Figure 4:**
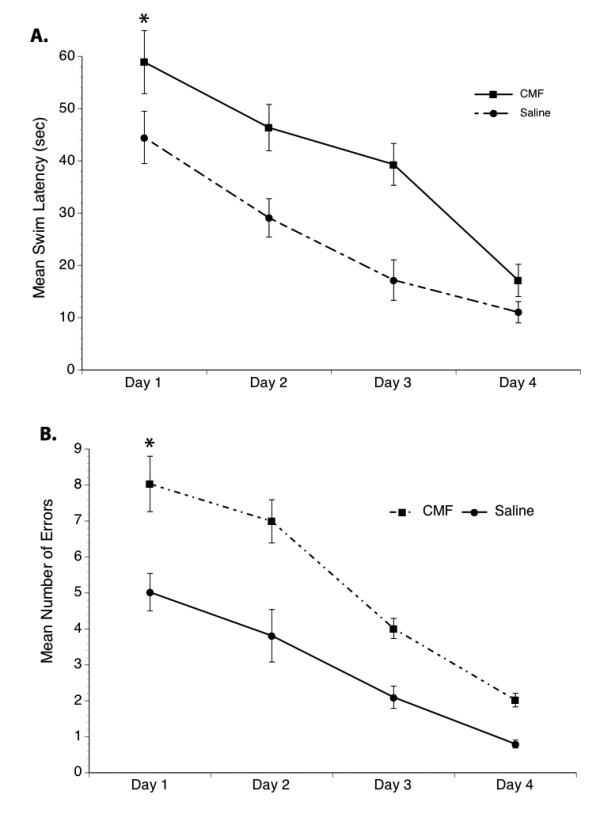
**Discrimination-Learning Test**. Significantly increased mean swim latency (A) and errors (B) were made by the CMF group compared to saline controls. However, all rats learned to perform the task. *p < 0.05.

In the discrimination-learning task a significant within subjects effect was seen for mean swim latency across the test days as all rats learned to perform the task efficiently over the 4 days of testing (Figure 4). An overall significant main effect of treatment was also seen. Specifically, chemotherapy-treated rats demonstrated longer mean swim latency compared to the saline-treated rats. Analysis of the number of choices made by the rats in locating the correct platform in the discrimination-learning task also showed an overall significant main effect of treatment. Saline-treated rats regularly choose P^+ ^(the correct goal) during the trials, while the chemotherapy-treated group made more errors by often choosing P^- ^(the incorrect goal) in the first 3 trial days (Figure 4). Although the chemotherapy-treated rats demonstrated longer mean swim latency and made more errors in the beginning days of the trial, they eventually learned to perform the task. In addition, a trend toward an interaction between treatment and time was seen. These data suggest that chemotherapy can induce transient impairment in the performance of simple learning and memory task but persistent cognitive dysfunction in the more complex test.

### Chemotherapy Decreases Cell Proliferation in the Hippocampus

**Figure 5:**
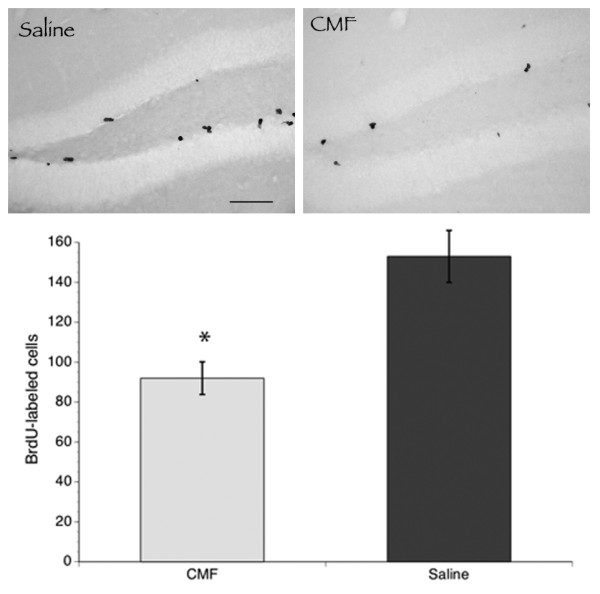
**Cell Proliferation**. Representative samples of BrdU labeling seen in the dentate gyrus of the hippocampal region in the CMF and saline groups (upper panel). Scale bar = 60 μm. CMF administration significantly decreased cell proliferation in the hippocampal region (lower panel). *p < 0.05.

The total number of BrdU-labeled cells in the dentate gyrus of the hippocampal region was quantified to determine cell proliferation. A significant main effect of treatment was seen in that the number of BrdU-positive cells per hippocampal volume decreased by approximately 20% in all rats subjected to the chemotherapeutic regimen compared to the saline group (Figure 5). These results suggest that there is a basal level of proliferative activity present in the mature nervous system evidenced by the presence of BrdU-positive cells in saline-treated rats but cytotoxic drugs can reduce cell proliferation.

### Chemotherapy Induces Histone Modifications in the Hippocampus

**Figure 6:**
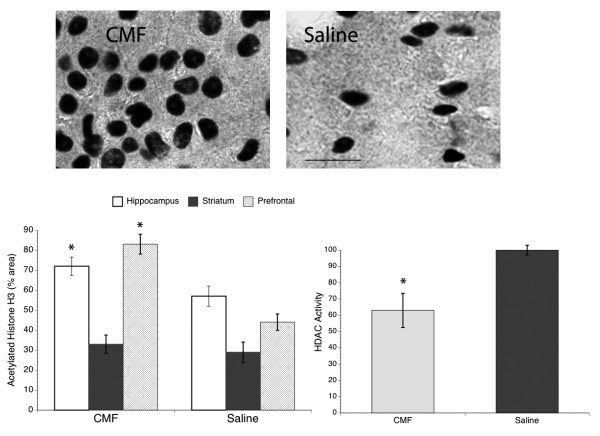
**Histone Modifications in the Hippocampus**. Representative samples of acetyl-H3 immunoreactivity in the hippocampus in the CMF and saline groups (upper panel). Scale bar = 40 μm. CMF administration significantly increased histone acetylation in the hippocampus, striatum, and prefrontal brain regions (lower panel, left). However, overall decreased in histone deacetylase activity was seen in the hippocampus of CMF-treated rats when compared to the saline group (lower panel, right). *p < 0.05.

Since the most studied mechanism of chromatin remodeling associated with learning and memory processes is histone modifications, we examined levels of histone acetylation and HDAC activity. Our results show significantly increased acetylation of histone H3 in the hippocampus and prefrontal cortex of chemotherapy-treated rats compared to the saline group, whereas no significant group changes seen in the striatum (Figure 6). Acetylation of histone H3 in the chemotherapy-treated rats is approximately 21% and 47% greater in the hippocampus and prefrontal cortex, respectively, when comparison to the saline group. In contrast, overall HDAC activity was significantly inhibited in the hippocampus of chemotherapy-treated animals by approximately 37% (Figure 6). These results suggest that the cytotoxic drugs can influence epigenetic modifications in certain regions of the brain.

## Discussion

In the present study using an animal model, we show learning and memory impairment following CMF administration, a drug combination that is widely used as chemotherapeutic agents in the treatment of human breast cancer. Since the behavioral tasks used in this study assess various aspects of learning and memory which can be dissociated and linked to different brain regions, the chemotherapy-induced deficits seen in both the spatial learning task and discrimination-learning tests provide insight into the areas that may be affected by the drugs. That is, the spatial memory evaluated in the water maze is a form of reference memory that depends on the functional integrity of the hippocampus while the ability to perform the discrimination-learning task depends on the functional integrity of the caudate nucleus and striatal structures. Since we demonstrate that CMF treatment resulted in impaired performance in both hippocampal- and non-hippocampal-dependent tasks, our results suggest that the adverse effects of these chemotherapeutic drugs are not limited to one region of the brain. Nevertheless, it is important to note that the effects of CMF on learning and memory while statistically significant are relatively small when compared to cognitive impairments that result from injury to the hippocampus and striatum. For instance, the learning and memory deficits seen in the water maze tests occurred only in the first 3 days of testing suggesting the transitory nature of the cognitive impairment. However, CMF-treated rats showed significant impairment in the discrimination-learning task throughout the test days when compared to the saline-treated rats suggesting the possibility that the water maze may lack sufficient complexity to detect subtle learning differences between the groups thus, behavioral performance will likely be affected by a "ceiling effect."

Our behavioral testing results parallel those of others that demonstrate cognitive impairment in rats and mice following administration of either methotrexate alone, cyclophosphamide alone, or the combination of methotrexate and 5-fluorouracil [12,13,15,16]. However, our data are at odds with others that showed no evidence of impaired cognitive performance in female rats when behavioral testing was conducted 7 weeks after cyclophosphamide or 5-fluorouracil administration [17]. The discrepancy in findings between our study and those of others may be attributed to the timing of behavioral testing; namely behavioral testing in our study was conducted two weeks after the last CMF injection when chemotherapy may still be readily available in the system, thus providing information on the short-term effects of the drugs. It is also possible that because of the longer treatment-testing interval in the Lee et al study [17], there may be some recovery of cognitive function in their animals compared to the rats tested in our study.

General activity levels and weight was also examined in the present study to ensure that behavioral differences between chemotherapy- and saline-treated rats are due to cognitive processes rather than drug-induced effects on performance ability. Although it is possible that drug-related side effects of fatigue and weight loss might have contributed to the cognitive impairment seen in the CMF-treated rats, this is unlikely for the following reasons: 1) while general activity decreased during the first 3 weeks of CMF administration it started to recover on the last week of treatment, and 2) even though daily food intake of the rats injected with CMF was significantly reduced for the first 3 days relative to the saline-treated animals, normal appetite rebounded beginning on day 4. In fact, one week after starting the CMF treatment rats that received the drugs actually had an overall increase in food intake compared to their pre-chemotherapy values. Thus, by the time behavioral testing was performed the transient changes in both general activity and body weight induced by CMF would likely have not contributed to the cognitive impairment seen in these rats.

The production of new neurons in the subgranular zone of the dentate gyrus is a well-characterized phenomenon in the mammalian brain that is related to learning and memory (for review see [21]). In the present study, we showed that CMF treatment significantly reduced the total number of proliferating cells in the dentate gyrus using BrdU-labeling and our data are in line with those reported by others that used the endogenous marker, Ki-67 [12,13]. These results are hardly surprising because both *in vitro *and *in vivo *data suggest a susceptibility of hippocampal regions to chemotherapeutic agents even at doses lower than those used in standard treatments [12,13,22]. For example, increased cell death and decreased cell division in the subventricular zone have been reported with administration of chemotherapeutic agents. Chemotherapeutic agents assumed to have minimal penetration into the CNS actually do enter the brain in small quantities (i.e., subclinical concentrations), and these quantities might be enough to cause toxicity and damage to neural progenitor cells [23]. Since the learning and memory impairments seen in our study are associated with decreased cell proliferation in the neurogenic area of the hippocampus, it is possible that chemotherapy-related cognitive dysfunction is caused by the disruption of endogenous adult hippocampal neurogenesis. Although it is possible that the decreased hippocampal cell proliferation seen in the CMF-treated rats does not have an effect on the differentiation and survival of the adult-born cells, in this study we primarily focused on potential influence of histone modifications on cell proliferation; however, the issue of CMF effects on the different phases of the neurogenesis process warrants further investigation in future studies.

Epigenetic mechanisms are often associated with learning and memory [24] and there is increasing evidence that histone modifications are in part responsible for regulating the process of adult hippocampal neurogenesis [25]. Here we demonstrate increased histone H3 acetylation and decreased HDAC activity in the hippocampus of the CMF-treated rats when compared to the saline-treated group. This is a surprising finding because histone acetylation is the post- translational modification on histones associated with enhanced learning and memory. A possible explanation for this unexpected finding is that the decreased HDAC activity may have contributed to the increased histone acetylation seen in the CMF-treated rats. It is possible that chemotherapeutic agents in general have HDAC inhibitor properties and this line of reasoning is supported by a recent study demonstrating that the mechanism of action of methotrexate is achieved through the downregulation of HDAC activity [26]. Moreover, our data that increased histone H3 acetylation in the hippocampus is not associated with enhanced cell proliferation is also surprising since epigenetic mechanisms have been implicated in neurogenesis. A possible explanation of this finding is that the method we used in the present study to measure acetylation/deacetylation only provides general information on histone modification. Examining specific epigenetic regulation of transcriptional pathways may be more valuable such as whether CMF-induced acetylation of the p53 pathway that leads to apoptosis [27] dampens the acetylation of the basic helix-loop-helix transcription factors that regulates cell proliferation; thus, these issues warrant further investigation in future studies.

## Conclusion

In sum, the present study provides evidence that anti-cancer drugs can adversely affect the self-renewal potential of neural progenitor cells and also chromatin remodeling in the hippocampus, which might be potential mechanisms in explaining chemotherapy-induced cognitive dysfunction. Our results also show the negative consequences of chemotherapy on brain function, apart from its potentially confounding physical side effects. Although more work is needed to establish the full extent of cognitive change following chemotherapy, our results on the degree of memory deficits seen in the CMF-treated rats parallel the human data in that clinical reports show cognitive deficits reported in chemotherapy-treated cancer patients to be typically mild to moderate in severity, and while they may impact day-to- day functioning, it is not necessarily apparent. Thus, the present results show that animal models can be useful in addressing the phenomenon of 'chemobrain.'

## Methods

### Subjects

Adult (4 months of age) female Wistar rats obtained from Harlan Laboratories (Madison, Wisconsin) were housed in pairs in a pathogen-free vivarium under controlled condition (temperature 22 ± 1°C and humidity 70 ± 5%) and a 14:10 hour light:dark cycle was maintained. All animals were housed in the same room so that temperature, humidity, and lighting conditions are similar for all groups. Animals had free access to food (regular rat chow) and water delivered through an automated and filtered system. Animals were also handled daily throughout the study so that they could get acclimated to the research personnel thereby decreasing stress. Experiments (see Study Design) started two weeks after arrival of the animals and all experimental protocols in this study were approved by the Institutional Animal Care and Use Committee and in accordance with the National Institutes of Health guidelines.

### Activity Measurement

Continuous activity monitoring was performed using a microtelemetry device (Data Science International, St. Paul, MN) to evaluate presence of fatigue due to chemotherapy. The device was implanted via laparotomy through a 1 cm incision in briefly anesthetized rats (gas anesthesia of 2.5% isofluorane and 30% oxygen mixture delivered through a cone). After telemetry implantation, rats were monitored every 10 minutes until fully awake then daily for presence of pain; if pain was observed (demonstrated as continuous scratching) then topical lidocaine was applied. The individual receiver boards that contain an infrared motion sensor placed under the floor of each cage continuously monitored general activity (frequency [Hz]). These data were fed into a peripheral processor connected to a computer, where they registered as activity counts and stored every 5 minutes. Activity counts were generated by any locomotion in the cage as detected by the infrared sensor from the implanted telemetry. The individual telemetry devices were calibrated before implantation and baseline activity level was collected for one week before the start of the chemotherapy protocol.

### Chemotherapeutic Regimen

Two weeks after recovery from minimitter implantation, rats were randomly assigned to either chemotherapy (n = 12) or saline control (n = 12) group. Rats in the chemotherapy group received the drug combination of cyclophosphamide (40 mg/Kg; Sigma-Aldrich, St. Louis, MO), methotrexate (37.5 mg/Kg; Wyeth Ayerst, Itasca, IL), and 5-fluorouracil (75 mg/Kg; Sigma-Aldrich, St. Louis, MO) dissolved in normal saline. Rats in the control group received normal saline of equal volume to control for the effects of stress induced by the injection. The dosages selected were based on our preliminary work, which showed that animals tolerated these doses with minimal weight loss or death. Both CMF and normal saline injections were given intraperitoneally once a week for a total of 4 weeks and rats were weighed every other day during the chemotherapeutic regimen. Rats were also monitored daily for other possible toxicity effects of chemotherapy (n = 0) such as apathy, excessive grooming, motor impairment, hair loss, and diarrhea.

### Cognitive Testing

Two weeks after the final CMF or saline injections, rats were tested in the water maze to evaluate cognitive impairment. The delay in behavioral testing was done to allow the animals to recover from the drug-induced fatigue that may confound the behavioral tests results. Both hippocampal and non-hippocampal learning and memory processes, were assessed. All testing were done approximately 2 hours prior to the onset of the dark cycle to ensure that it is close to the rats' active period.

Spatial learning and memory (acquisition and recall), tasks sensitive to hippocampal dysfunction were examined using the water maze task. The water maze apparatus consisted of a circular tub made of galvanized steel measuring 1.52 m in diameter; and the interior surface was painted white. The use of a large tub decreased the probability that the rats will find the goal/platform by chance. During testing, the tub was filled with tepid water (22 ± 2°C) and made opaque by the addition of powdered milk. An inverted white flowerpot, submerged 2 cm beneath the water's surface served as the goal/platform and the opaqueness of the water enabled the goal/platform to be concealed. Extramaze cues, such as overhead lighting, windows and room noise were held constant during testing. The pool was divided into four quadrants of equal surface area and the starting locations for testing were assigned north, south, east, and west. The goal/platform was located in the middle of the southeast quadrant approximately 22 cm from the pool rim. The day before actual testing started, rats were allowed a habituation swim for 10 seconds without the platform. The habituation swim and consistent water temperature throughout the test days were necessary to minimize animal stress during water maze testing. Animals received four trials a day for four consecutive days. A different starting point was used on each of the four daily trials and the order of starting points was random. If the rat failed to find the hidden platform within 3 minutes, they were guided to the platform and given a swim latency score of 180 seconds. The animals were allowed to stay on the platform for 20 seconds then towel-dried until the next trial. A minimum of two minutes was used between trials to provide a rest period for the animals and avoid "practice effect." During the trials, swim latency (time to reach the platform) and the path taken by the animals to reach the platform were recorded by a video camera connected to an image analyzer (Water Maze System Version 4.20, Columbus, OH) and these data were used to assess performance in the water maze task. In addition, swimming speed (path length/swim latency) was used to assess the motoric activity of the rats in performing the task. Black shoe polish was applied on top of the animals' head to facilitate video camera tracking as rats swim in the water maze. On the fifth day, a probe trial was performed wherein the goal/platform was removed from the pool. In addition, rats were tested in a cued trial for one day (4 trials) following the water maze task where they were allowed to swim in the tub to locate a visible pole attached to the goal. The cued trial was performed to control for potential visual problems that may influence performance in the water maze.

The rats also received discrimination-learning test after two days of rest following the cued trials. The discrimination-learning task is sensitive to dysfunction involving the striatum. In this task the rats had to discriminate between black and white visible goals to find the hidden platform and all extra-maze cues in the room were covered. The goal painted white was placed on top of the hidden platform to provide escape (P^+^) from the water (located in the southeast quadrant); whereas the other one painted black was floating (P^-^) and not able to offer sufficient buoyancy to support the rat (located in the southwest quadrant). Both visible goals were placed 8 cm above the water level. For this task, the additional measure obtained was the number of correct choices of P^+ ^compared to P^- ^since the aim was to train the rats to avoid P^-^.

### Cell Proliferation

The thymidine analog Bromodeoxyuridine (BrdU; Chemicon, Temecula, CA) was used to label proliferating cells. BrdU incorporates into the genetic materials on mitotic division within 2 hours after injection, after which it can be detected immunohistochemically in the daughter cells [28]. BrdU was dissolved in 0.9% sterile NaCl and filtered at 22 μm. The resulting solution was injected at 100 mg/kg intraperitoneally in all rat groups. Injections were given 4 hours prior to euthanasia.

### Tissue Preparation

All rats were euthanized using CO_2 _inhalation, the brains removed, cut in half sagitally and immediately placed in liquid nitrogen until processed. Half of the brain was used for immunohistochemistry (detection of cell proliferation and histone acetylation) while the other half was used for determination of histone deacetylase (HDAC) activity. The half used for immunohistochemistry was fixed in 4% paraformaldehyde in 0.1 M phosphate buffer (pH 7.3) overnight then cryoprotected before sectioning.

### Immunohistochemistry

The fixed brains were sectioned at 30 μm thickness using a cryostat. Tissue sections were obtained covering the entire hippocampal region in its rostro-caudal extension and the free-floating section method was used for immunohistochemistry to examine histone modifications and hippocampal cell proliferation. For detection of BrdU-labeled nuclei, DNA was denatured to expose the antigen before incubation in anti-BrdU primary antibody. Briefly, free-floating sections were pretreated in 50% formamide/50% 2xsaline-sodium citrate buffer (SSC) at 65°C for 2 h, rinsed in 2xSSC, and then incubated in 2 N HCl at 37°C for 30 min. Tissues were then rinsed in borate buffer (pH 8.5) for 15 minutes and placed in 0.6% H2O2 in Tris-buffered saline (TBS) for 30 minutes to block endogenous peroxidase, followed by several rinses in TBS (pH 7.5). Tissues were then placed in TBS/0.1% Triton X-100/3% donkey serum (TBS-TS) for 1 hour followed by incubation with anti-BrdU primary antibodies at a concentration of 1:400 (monoclonal mouse; Boehringer Mannheim; Indianapolis, IN) in TBS-TS overnight at 4°C. The following day, the primary antibody was detected using biotinylated immunoglobulin G (IgG) donkey anti-mouse secondary antibodies (Vector Laboratories; Burlingame, CA) at a concentration of 1:200 for 2 hours. Tissues were then rinsed in TBS and incubated in avidin-biotin complex (ABC kit; Vector Laboratories) for 1 hour at room temperature. Immunoreactions were visualized by treatment of section with hydrogen peroxide and 3,3'- diaminobenzidine tetrahydrochloride in Tris buffer (pH 7.3). After thorough rinsing, the tissue sections were mounted on gelatin-coated slides and dried, and coverslips were applied. To minimize intergroup and interbrain staining variability and to ensure reproducibility of results, tissues from all experimental groups were run simultaneously and under identical conditions.

The total number of BrdU-positive cells in the granule cell layer and its corresponding sample volume were determined in 8 coronal sections, 240 μm apart, using the optical disector method (StereoInvestigator, MicroBrightfield, Colchester, VT). Briefly, each section was examined at a magnification of 40x, and an unbiased counting frame was positioned randomly across the dentate gyrus area. The 1st focal plane (i.e., the top of the tissue section where cells came into focus) was identified, and cells in this field of view were disregarded. A focal plane (approximately 3 μm apart) was then gradually passed through each section by adjusting the focus of the microscope slowly, and the labeled cells encountered while focusing through the section were counted. The number of labeled cells was related to the number of sections counted and was multiplied by the reference volume to provide an unbiased estimation of the total number of BrdU-positive cells. Reference volume in the dentate gyrus was obtained using the Cavalieri principle, wherein the granule cells were counted at random systematic sampling points superimposed onto the image projected on the computer. The reference volume was the product of the sum of the number of points that fell within the boundaries of the granular layer and the mean post- processing thickness of Nissl-stained sections. The section thickness of 30 μm (microtome setting) was used because it was assumed that the net error by using the whole-section thickness for the volume was smaller than the error introduced by measuring the postprocessing section thickness on each slide and counting in a fixed fraction of it.

For detection of histone acetylation, tissues were first sequentially treated with 0.3% hydrogen peroxide in PBS for 30 minutes then rinsed with 0.1 M phosphate buffered saline (pH 7.3) and placed in the blocking solution of 3% serum, 0.1% Triton-X, and 1% bovine serum albumin for 1 hour. After blocking, tissues were washed in phosphate buffered saline (PBS) followed by incubation for 24 hours at 4°C in rabbit anti-acetyl-H3 (1:1000; Upstate Cell Signaling, Bellirica, MA) with gentle agitation. Anti-acetyl-H3 recognizes histone acetylation at the Lys9 and Lys14 residues. The primary antibody was detected using preadsorbed biotinylated IgG secondary antibodies (1:200, Vector Laboratories, Burlingame, CA) for 1 hour at room temperature. The tissues were then washed and incubated in avidin-biotin complex (ABC kit, Vector Laboratories, Burlingame, CA) for one hour at room temperature. Immunoreactions were visualized by treatment of tissue sections with hydrogen peroxide and 3,3'-diaminobenzidine tetrahydrochloride (DAB) in Tris buffer (pH 7.3). After thorough rinsing, the tissue sections were mounted on gelatin-coated slides, dried, and coverslipped. Tissues from all experimental groups were run simultaneously and under identical conditions to ensure reproducibility of results. In addition, a pre-dilution test was done to ensure specificity of the antibody and negative controls, involving deletion of the primary antibody, were used to rule out any nonspecific interactions. Quantification of histone H3 changes in the hippocampus, striatum, and prefrontal cortex was determined by the surface area covered by anti-acetyl-H3 immunoreactivity using an area-fractionator grid defined by the StereoInvestigator (MicroBrightfield, Colchester, VT) computerized analysis system as previously described [29].

### Histone Deacetylase (HDAC) Activity

To determine histone deacetylation, HDAC activity was measured from the total cell lysate after whole hippocampal tissues were homogenized using a nuclear extraction kit (Sigma, St. Louis, MO). Total HDAC activity (class I and II HDACs) was determined according to the manufacturer's instructions for the colorimetric HDAC activity assay kit (BioVision Research, Mountain View, CA) by measuring the deacetylation of acetylated lysine side chains. The optical density (OD) of the samples was measured using an ELISA plate reader at 405 nm (Spectra MR; Dynex Technologies, Chantilly, VA). The results were calculated as OD per milligram of protein and then converted to percentage of control.

### Statistical Analysis

The SAS general linear model (SAS Institute, North Carolina) procedures for one-way analysis of variance (ANOVA) were used to examine effects of experimental conditions (chemotherapy vs. saline groups) on epigenetic modifications (histone acetylation and HDAC activity). Repeated measures ANOVA were used to examine chemotherapy effects on weight loss, activity, and behavioral performance to determine differences in latency, path length, swimming speed, exploration time, and discrimination ratio. All error bars represent ± standard error of the mean (SEM) of the sample size used in the study.

## Competing interests

The authors declares that they have no competing interest.

## Authors' contributions

TLB conceived of the study and participated in its design and coordination, performed the BrdU protocol and statistical analyses, and drafted the manuscript. JW carried out the immunohistrochemistry and assay procedures as well as behavioral testing protocols. All authors read and approved the final manuscript.
